# Injury epidemiology, patterns, and risk factors in youth and adolescent badminton players: a PRISMA-ScR–guided scoping review

**DOI:** 10.3389/fspor.2026.1883485

**Published:** 2026-07-23

**Authors:** Payal Vasant Dhawale, Vishnu Vardhan, Sarath Babu, Ayyappan Jayavel

**Affiliations:** 1 Ravi Nair Physiotherapy College, Datta Meghe Institute of Higher Education and Research (DMIHER), Wardha, India; 2 MGM Institute of Physiotherapy, Chhatrapati Sambhajinagar, affiliated to Maharashtra University of Health Sciences, Nashik, India; 3 Department of Cardiovascular & Respiratory Physiotherapy, Ravi Nair Physiotherapy College, Datta Meghe Institute of Higher Education and Research (DMIHER), Wardha, India; 4 SRM College of Physiotherapy, SRM Institute of Science and Technology, SRM Nagar, Kattankulathur, Chennai, India

**Keywords:** adolescents, badminton, epidemiology, injury prevention, sports injury, youth

## Abstract

**Introduction:**

Badminton is the most popular sport among adolescents across the world. Injury-related research focusing specifically on this age group remains limited. Much of the existing literature combines adolescent players with broader athletic populations, making it difficult to understand injury patterns unique to youth and adolescent badminton players. This review examined the available evidence related to injury prevalence, incidence, anatomical distribution, injury characteristics, and potential risk factors in youth and adolescent badminton players (aged 7–22 years, with some studies including broader age ranges). It also aimed to highlight areas where current knowledge of injury prevention remains incomplete. This is the first scoping review to apply the Preferred Reporting Items for Systematic Reviews and Meta-Analyses extension for Scoping Reviews (PRISMA-ScR) methodology to youth badminton injury literature, enabling systematic mapping of evidence gaps and informing future research priorities in this area.

**Methods:**

This scoping review followed the Arksey and O'Malley framework and the PRISMA extension for scoping reviews. A comprehensive English-language search was conducted in June 2025 using PubMed, Scopus, the Cochrane Library, Google Scholar, and reference list screening. Ten observational studies met the eligibility criteria. The studies varied in design, including prospective, retrospective, and cross-sectional approaches. Injury data were collected during training, competition, or both, and different injury definitions were used, including pain- or symptom-based, time-loss, and medical attention. Exposure measurements also differed across studies, with reports based on training hours or player exposure. Owing to these methodological differences, a meta-analysis was not feasible, and the findings were summarized descriptively without formal quality appraisal, consistent with the scoping review methodology.

**Results:**

Ten observational studies were included in this review. The included studies showed substantial variations in study design, settings, injury definitions, and exposure measurements. Injury prevalence ranged from 18.1% in Malaysian national-level players to 33.87% in elite Sri Lankan junior players, although differences in injury definitions and study populations limit direct comparisons between studies. Studies using pain- and symptom-based surveillance, which captures minor symptoms that do not require medical attention, have reported a prevalence of musculoskeletal complaints exceeding 60% in larger Japanese youth badminton cohorts.

**Conclusion:**

Injuries are common among youth and adolescent badminton players. Limited evidence from two prospective studies suggests that injury risk may increase during mid-to-late adolescence, and higher injury rates have been reported among elite-level players with greater training exposure. Variations in injury definitions, surveillance approaches, and exposure measurements across studies make comparisons difficult and reduce consistency within the current evidence base.

## 1 Introduction

Badminton is a widely embraced sport among youth worldwide, with particularly high levels of participation in Asia and Europe. Countries such as China, India, Indonesia, and Malaysia have established structured training programs and competitive pathways that actively promote youth engagement in the sport. These systems not only nurture talent but also contribute to the sustained popularity and development of badminton at the grassroots and elite levels across these regions (1–4). and Indian youth players have achieved high rankings in the Badminton World Federation (BWF) Junior Rankings, showing badminton's growing prominence in India globally (4).

In badminton, repetitive high-intensity actions such as lunging, jumping, rapid acceleration-deceleration, multidirectional cutting, and overhead strokes significantly impact biomechanical stress on the musculoskeletal system. The combination of high biomechanical demands and physiological vulnerabilities in adolescence, including rapid growth, hormonal changes, physeal vulnerability, and neuromuscular immaturity, increases the susceptibility to musculoskeletal injuries. The evidence highlights the importance of neuromuscular training, biomechanical assessment, and equipment considerations in mitigating injury risk in youth and adolescent badminton players (5–10). Traditional youth sports pathways involve high training volumes and year-round participation. In badminton, this includes early sport-specific training with limited movement variations. These practices often have congested schedules and insufficient recovery, which increases repetitive loading and may lead to overuse injuries (11, 12). During this phase, rapid skeletal growth exceeds muscle and tendon adaptation, causing muscle-tendon imbalances and reduced neuromuscular control (13).

Despite the growing involvement of adolescents in competitive badminton, there is a notable absence of comprehensive, badminton-specific synthesis that addresses injury patterns and risk factors within this demographic. Although previous reviews have examined badminton injuries across mixed-age or adult populations (14), no extensive synthesis has focused exclusively on youth and adolescent players, who possess distinct physiological and training-related vulnerabilities. This gap highlights the need for targeted analyses that integrate current evidence relevant to this group.

This scoping review systematically investigated the existing scientific literature on injuries among youth and adolescent badminton players. The guiding research question is: “What is the current evidence on the prevalence, types, anatomical locations, and risk factors of injuries in youth and adolescent badminton players?” By synthesizing the available evidence, this review aims to:

- To describe the patterns and characteristics of injuries, including common anatomical sites and injury types, in youth and adolescent badminton players.
- To identify demographic, training-related, and sport-specific risk factors associated with these injuries.
- This study suggests directions for future research and targeted injury prevention strategies for coaches, parents, and sports organizations.

Although adolescence is broadly defined as 10–19 years of age, the available literature on badminton injuries frequently includes broader age ranges spanning children, adolescents, and adults. Accordingly, this review included studies involving participants aged 7–22 years, as well as studies with wider age ranges when age-stratified data for adolescents were reported or when most participants were within the target age range. The findings were synthesized with careful consideration of the participants' age and study context. Therefore, the review population is best described as youth and adolescent badminton players, based on evidence from studies that occasionally included broader age groups. The findings will provide valuable insights for sports medicine practitioners, physiotherapists, coaches, and policymakers, supporting evidence-based approaches to enhance the safety, training, and performance of youth and adolescent badminton players in the future.

### 1.1 Rationale for the review

This scoping review used the PRISMA-ScR methodology to systematically map the evidence, including heterogeneous study designs and injury definitions, without a formal quality appraisal. This approach, which has not been applied to the youth badminton injury literature, enables comprehensive identification of knowledge gaps and informs future research priorities. This review addresses this gap by synthesizing evidence on injury prevalence, types, anatomical distribution, and risk factors among youth and adolescent badminton players. Although badminton is practiced at youth levels, epidemiological data on adolescents are limited and inconsistent, impeding the characterization of injury trends during this developmental stage. Badminton is a high-intensity sport involving accelerations, lunges, jumps, and multidirectional changes in direction, causing joint loading and repetitive tissue stress (14). During adolescence, skeletal maturation and neuromuscular development may affect susceptibility to injuries. By including adolescents and age-stratified youth and young adult populations, this review outlines the patterns of adolescent injuries. The findings aim to inform developmentally appropriate prevention strategies, support evidence-based decisions among coaches and sports medicine professionals, and guide research to promote safe and sustainable badminton participation.

## 2 Methods

We adhered to the methodological framework for scoping reviews outlined by Arksey and O'Malley (15), which consists of five stages: (1) identifying the research question, (2) identifying relevant studies, (3) study selection, (4) charting the data, and (5) collating, summarizing, and reporting results. Steps 4 and 5 were combined to improve efficiency without compromising the quality of analysis.

### 2.1 Protocol registration

The review was reported in accordance with the PRISMA-ScR guidelines to ensure transparency and completeness of reporting. The review protocol was not prospectively registered in a public repository. However, the methodology was predefined prior to the study initiation and implemented in accordance with the established scoping review guidance to support transparency and reproducibility.

### 2.2 Research question

As outlined above, this scoping review was guided by the following research question: “What is the current evidence on the prevalence, types, anatomical locations, and risk factors of injuries in Youth and Adolescent Badminton Players?” The aim of the subsequent steps was to map existing research on injury patterns and determinants without formal quality appraisal to identify knowledge gaps and inform future research.

### 2.3 Identifying relevant studies

To identify relevant studies, a comprehensive literature search was conducted in June 2025 across three electronic databases: PubMed, Scopus, and the Cochrane Library. Systematic searches were performed in each database using adapted search strategies to maximize sensitivity and capture all relevant studies. No publication date restrictions were applied to ensure a comprehensive search that captured all studies published in English until the search date was applied. The search strategy was developed using the Population–Concept–Context framework, as recommended for scoping reviews. The Population comprised badminton players relevant to adolescents, the concept focused on injury patterns and injury-related risk factors, and the context was competitive and training-based badminton settings.

The search was conducted in a two-step process as follows.

Primary search: Initial searches were performed in each database using predefined key terms related to badminton, youth and adolescent players, sports specialization or intensive training, and sports injuries. Searches were limited to the title and abstract fields, where applicable.

Secondary search: A refined search was conducted using additional keywords identified from articles retrieved during the primary search to ensure that variations in terminology related to training characteristics and injury outcomes were not missed.

**Figure 1:**
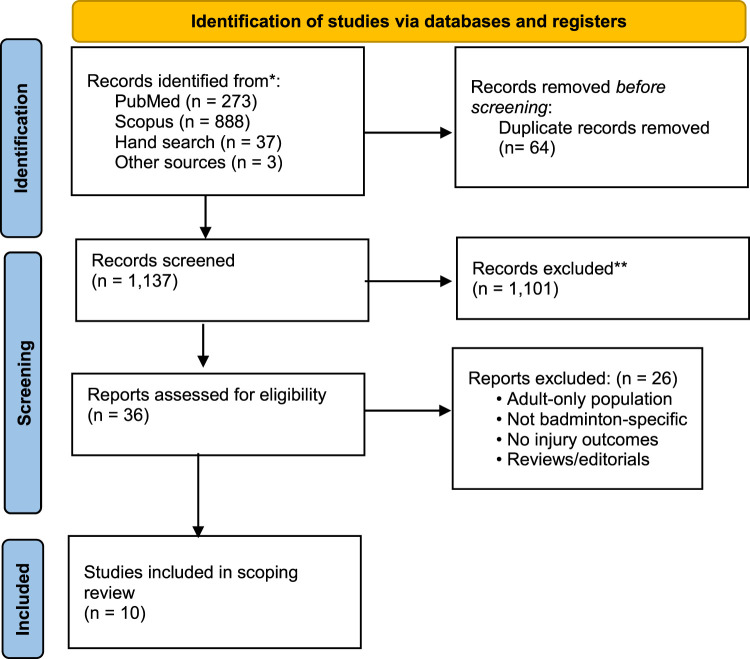
PRISMA flowchart. Adapted from the PRISMA 2020 flow diagram template (34).

In addition to this two-step process, supplementary searches were conducted by screening the reference lists of the included studies and using Google Scholar to identify additional relevant publications. The final search strategies were adapted according to the requirements of each database, and the details are provided in Table 1. The study selection process is illustrated in Figure 1.

**Table 1:** Search strategy and database yield.

Component	Search terms used
Search strategy	(“badminton”[MeSH Terms] OR “badminton”[All Fields] OR “badminton player*”[All Fields] OR “racquet sports”[All Fields]) AND (“adolescent”[MeSH Terms] OR “adolescent”[All Fields] OR “youth”[All Fields] OR “young players *”[All Fields] OR “school-aged”[All Fields]) AND (“injuries”[MeSH Terms] OR “injuries”[All Fields] OR “injury prevalence”[All Fields] OR “injury incidence”[All Fields] OR “risk factors”[All Fields])
Controlled vocabulary	Medical Subject Headings (MeSH) and database-specific indexing terms
Boolean operators	Population AND Concept AND Context
Search fields	Title and Abstract (where applicable)
Language limits	English
Date limits	None (from database inception to June 2025)
Databases searched	PubMed (*n* = 273 records), Scopus (*n* = 888 records), Cochrane Library (screened; no eligible records identified)

### 2.4 Study selection

All records identified through database searches and additional sources were imported into a reference management system, and duplicate records were removed before screening. After deduplication, only records retrieved from PubMed and Scopus that contributed to the eligible studies for screening were included. All records retrieved from PubMed, Scopus, and the Cochrane Library were exported to Rayyan (Qatar Computing Research Institute), an online systematic review management platform, for duplicate removal and screening of records. Titles and abstracts were screened by a single reviewer according to the predefined inclusion and exclusion criteria. Full-text articles were subsequently assessed using eligibility criteria.

To enhance methodological rigor and minimize selection bias, eligibility criteria were established *a priori*, and screening decisions were documented within the Rayyan platform to ensure transparency and traceability. Ambiguous or borderline studies were re-evaluated against predefined criteria before final inclusion to maintain consistency in study selection. for relevance to badminton-related injuries, with particular emphasis on studies involving youth and adolescent badminton players.

Full-text articles were retrieved for records that met the inclusion criteria or for which eligibility could not be clearly determined based on the title and abstract. Full-text screening was conducted using the predefined eligibility criteria given in Table 2. Studies were included if they reported injury prevalence, incidence, injury patterns, anatomical distribution, or injury-related risk factors in badminton players, with specific relevance to the adolescent population.

**Table 2:** Inclusion and exclusion criteria for study selection.

Domain	Inclusion criteria	Exclusion criteria
Population	youth and adolescent badminton players; studies primarily targeting adolescents (age not strictly specified)	Studies involving only adult or veteran badminton players and not reporting data relevant to youth or adolescent participants.
Sport	Badminton (including school, club, district, state, national, or elite junior levels)	Studies focusing on other sports or mixed-sport populations where badminton data could not be isolated
Exposure/focus	Intensive training, single-sport participation, or training-related load	Studies not addressing, training exposure, or related participation characteristics
Outcomes	Musculoskeletal injuries, pain, injury prevalence, injury incidence, injury patterns, injury severity, or injury risk factors	Studies not reporting injury- or pain-related outcomes
Study design	Observational studies (cross-sectional, cohort, prospective, retrospective), surveillance studies	Case reports, editorials, commentaries, letters, conference abstracts without full text
Language	Studies published in English	Non-English publications
Publication type	Peer-reviewed journal articles	Theses, dissertations, books, gray literature
Publication Date	No restrictions applied (all studies available up to June 2025)	—
Accessibility	Full-text articles available	Full text not accessible

Studies were excluded if they focused exclusively on adult populations, were not badminton-specific, did not report injury-related outcomes, or were review articles, editorials, or conference abstracts. Any uncertainties regarding study eligibility were resolved through discussion among reviewers. Finally, 10 studies met the inclusion criteria and were included in the descriptive synthesis process.

### 2.5 Charting data and collating, summarizing, and reporting results

The included studies were systematically organized into a summary Table 3, in which the key characteristics of each study were collected. The extracted information included the reference (first author and year), country, study design, participant characteristics, sample size, competitive level, injury definition, injury assessment method, injury type, anatomical location, and reported injury outcomes.

**Table 3:** Characteristics and injury outcomes of included studies (*n* = 10).

Sr. no	Author (Year)	Country	Sample/age	Study design	Level of play	Injury type(s)	Exposure denominator availability	Setting	Outcome measure type
1	Zhou et al. (2025) (1)	Japan	711 badminton players:Age range 7–22 years	Retrospective	National competitive level	Badminton-related musculoskeletal injuries	Per 1,000 training-hours (calculated using Poisson distribution)	National competitive badminton tournaments (3 randomly selected locations for 7–12 years; 10 locations for 13–17 years; 2 universities for 18–22 years)	Injury incidence rate per 1,000 training-hours; Injury prevalence (%)
2	Zhou et al. (2023) (16)	Japan	366 pre-adolescent and youth and adolescent badminton players aged 7–12 years	Questionnaire survey (cross-sectional)	Pre-adolescent and adolescent players; members of Japan Schoolchildren Badminton Federation attending national tournament games	Pain, acute injury, gradual-onset injury	Per 1,000-training-hour exposure	National tournament games at three randomly selected locations in Japan (2019)	Pain incidence calculated as pain rate per 1,000-training-hour exposures with 95% confidence intervals
3	Miyake et al. (2022) (17)	Japan	133 national-level badminton players (junior high school, high school, and university)	Prospective longitudinal study	National-level tournaments	Trauma and Overuse	Players Exposures (AEs)—defined as one participation in training or match play by an players	Gymnasium during training and match play	Not explicitly stated in document
4	Suryanto et al. (2022) (18)	Indonesia	128 junior badminton players s; Age categories: <11 years (U-11), <13 years (U-13), <15 years (U-15), <17 years (U-17), <19 years (U-19)	Retrospective cohort study	Elite/National Premier Circuit level	Acute injuries and Chronic/Overuse injuries	Incidence proportion (18%) and clinical incidence (18.8 per 100 players s)	National Premier Circuit tournament in West Java, Indonesia (5 consecutive days in June 2019)	Injury rates (IR) per 1,000 players exposures (AEs) and IR per 1,000 h
5	Zhou et al. (2022) (19)	Japan	510 elementary school-aged badminton players (7–12 years old)	Questionnaire study	Elementary school-aged badminton players (recreational/competitive)	Pain (648), Traumatic injuries (210), Overuse injuries (186)	Per 1,000 players-hours of exposure	Training and match play	Incidence proportion and clinical incidence (per 100 players s)
6	Senadheera et al. (2021) (20)	Sri Lanka	62 badminton players; Age range 8–17 years	Descriptive cross-sectional study	Elite junior players competing at national level tournaments	Traumatic (acute) injuries and overuse (chronic) injuries	Not explicitly stated in study	Kandy district, Sri Lanka; Practice sessions and competition settings	Injury incidence rate per 1,000 players-hours; Proportions by anatomical region; Injury incidence percentages
7	Zhou et al. (2021) (21)	Japan	611 badminton players (260 males, 351 females) aged 7–12 years	Cross-sectional study using self-reported questionnaire	National tournament participants	Shoulder pain, lower back pain, knee pain; categorized as traumatic injuries (acute onset) and gradual-onset	Expressed as per 1,000 players-hours of exposure	Japan Schoolchildren Badminton Federation national tournament	Prevalence rates, injury location distribution, injury type classification, temporal patterns (competition vs. practice), severity classification
8	Kaldau et al. (2021) (3)	Multi-national study; 164 players from 32 countries from all continents	164 of 436 tournament competitors 15–19 years	Cross-sectional study according to STROBE guidelines	World elite junior badminton players aged 15–19 years	Tendinopathies (14%), fractures (10%), sprains (10%), stress fractures (11%)	Not available	2018 World Junior Badminton Championships	Incidence rate per 1,000 players-hours of exposure; Odds ratios (OR) with 95% confidence intervals (CI) from multivariate logistic regression analysis
9	Miyake et al. (2016) (22)	Japan	133 players (94 male, 39 female) aged 13–20 + years from junior high school to university	Prospective epidemiological longitudinal survey	national tournament-level badminton players	Trauma or overuse	IR (1,000 h) and IR (1,000 players exposures)	Gymnasium	Prevalence of significant injuries and current musculoskeletal symptoms
10	Goh et al. (2013) (2)	Malasiya	58 competitive youth badminton players (13–16 years old)	Prospective Observational study	Elite youth national level	Traumatic, Overuse, Indetermine	1,000 training hours	Training, competition	Injury rate (IR) per 1,000 h; IR per 1,000 players exposures (AE)

Following data charting, an overview of the included evidence was generated to describe the current state of research on injuries in youth and adolescent badminton players and identify existing knowledge gaps. Given the heterogeneity in study designs, injury definitions, and outcome measures, the results were collated and summarized accordingly.

A narrative synthesis was conducted to integrate the findings across the studies, focusing on the injury prevalence, commonly affected anatomical regions, and reported injury patterns. The results were interpreted within the broader context of youth and adolescent racquet sports research to provide meaningful insights for clinicians, coaches, and researchers. Reporting followed the PRISMA-ScR guidelines, ensuring a transparent and structured presentation of the evidence.

#### 2.5.1 Injury definitions

Injury definitions differed considerably between studies because of the different study designs and monitoring methods used. Mentioned in Table 4 therefore, direct comparisons should be interpreted with caution. Most studies broadly defined injury as a physical complaint or musculoskeletal condition associated with badminton participation, irrespective of the need for medical attention or time-loss due to injury. Several studies have adopted inclusive definitions encompassing pain, discomfort, overuse symptoms, and acute traumatic injuries.

**Table 4:** Injury definitions.

Sr. no	Author (Year)	Injury definition type	Definition details	Verbatim definition
1	Zhou et al. (2025) (1)	Time-loss and/or medical care	Any somatic complaint resulting in: (1) inability to continue current match/training; (2) inability to attend next match/training; and/or (3) requirement for medical care	Any somatic complaint that resulted in one or more of the following three situations: (1) inability to continue the current badminton match or training session; (2) inability to attend the next match or training session; and/or (3) requirement for medical care regardless of the likelihood of absence from a match or training session.
2	Zhou et al. (2023) (16)	According to International Olympic Committee guidelines	Any painful physical discomfort (ache or soreness in anatomical regions, without or with radiating pain) with sustained sports capability including: (1) ability to continue badminton training/match; (2) participation in next scheduled training/match without time-loss; (3) no need for medical care	Any physical discomfort that caused one or more of the following: (1) having to immediately stop present training/match; (2) being absent from following training/match; and/or (3) need for medical attention regardless of missing training/match
3	Miyake et al. (2022) (17)	Physical complaint with abstinence requirement	Physical complaint occurring during play, causing abstinence from subsequent training or match play, requiring medical attention from team physical therapist	A physical complaint that occurred during play, caused abstinence from subsequent training or match play, and required medical attention from a team physical therapist at the scene
4	Suryanto et al. (2022) (18)	Musculoskeletal	Any musculoskeletal symptoms that occurred during the tournament	Musculoskeletal injuries were defined as any musculoskeletal symptoms that developed during the tournaments. Reports of pre-existing injuries or injuries that were not fully rehabilitated were excluded from the study.
5	Zhou et al. (2022) (19)	Traumatic (acute onset) and Overuse (chronic without trauma)	Injury defined as any physical complaint sustained during training or match play resulting in: 1) inability to complete current match or training; 2) absence from subsequent training or matches; 3) injury requiring medical attention	An injury was defined as any physical complaint sustained during training or match play resulting in at least one of the following: 1) inability to complete the current match or training, 2) absence from subsequent training or matches, or 3) injury requiring medical attention.
6	Senadheera et al. (2021) (20)	Operational/Functional definition	Any kind of injury, pain or physical damage that occurs during playing badminton or training and leads to player being unable to fully participate in training or match play	"Badminton injury was defined as any kind of injury, pain or physical damage that occurs during playing badminton or training and lead to player being unable to fully participate in training or match play"
7	Zhou et al. (2021) (21)	Two definitions: Pain and Injury	Pain: Any physical painful complaint or discomfort during sustained badminton performance. Injury: Physical complaint sustained during training or match play requiring one or more of: (1) immediate cessation of activity, (2) absence from subsequent training/matches, (3) medical attention with time-loss	Pain was defined as any physical painful complaint or discomfort with sustained badminton performance. An injury was defined as any physical complaint sustained during training or match play resulting in one or more of the following three judgement criteria: (1) the need to stop the current training or match immediately, (2) absence from subsequent training or matches, and (3) the need for medical attention with time-loss injuries.
8	Kaldau et al. (2021) (3)	Significant injuries—operational definition	Injury lasting minimum 30 days causing reduction in training capacity and/or absence from competition	Any physical complaint sustained by a player that results from a badminton match or training, regardless of the need for medical attention or time-loss from badminton activities. A significant injury was defined as an injury lasting a minimum of 30 days, causing the player to have a reduction in training capacity and/or absence from competition.
9	Miyake et al. (2016) (22)	The injury records included the day of injury, injury type (trauma or overuse), and number of days between the injury and the player's return to practice or match. Classification-based (Hagglund et al. model)	Injury defined as event causing player to drop out of practice or match; required medical attention from dedicated PT	“An injury is defined as an event dropout from practice or match. Injuries were those that required medical attention”
10	Goh et al. (2013) (2)	Clinical Assessment type	Requires medical attention	Injury that appeared to be connected with badminton training or match 1. handicapped player during play 2. required special treatment (medical treatment) to play 3. made play impossible 4. affected the availability for selection of a player

The studies did not have the same criteria for defining injury for younger or pre-adolescent players; some used pain- or symptom-based definitions, while in some, injuries were identified through self-reported pain without requiring time-loss or medical consultation (16, 21). As presented in Table 4, these studies mainly used questionnaire-based methods and examined the prevalence of pain or symptoms associated with training and competition exposure.

The other two studies defined time-loss and/or medical attention (1, 18). In these studies, injuries were recorded only when participation was restricted, or medical care was required. Most of these investigations collected information prospectively or through medical records, which also made it possible to report injury incidence rates more clearly.

Some studies have further categorized injuries based on their mechanism and onset, distinguishing between acute injuries resulting from a single identifiable event and overuse (chronic) injuries that develop gradually due to repetitive loading (20, 22). Additionally, several studies have classified injury severity using categories such as minimal, mild, or slight, based on the degree of functional limitation and/or time-loss (22).

Overall, this highlights the substantial heterogeneity in injury definitions, data sources, and exposure denominators across studies. While some relied on pain-based or self-reported measures, others used time-loss or medically diagnosed criteria, resulting in differences in case identification and injury severity classification. This variability limits the comparability of injury prevalence and incidence estimates across studies. Standardized approaches to injury surveillance, such as those recommended by the International Olympic Committee, emphasize consistent injury definitions, exposure measurements, and reporting frameworks. The limited adoption of such standardized methodologies likely contributes to the observed methodological heterogeneity (23).

#### 2.5.2 Study characteristic related to injury surveillance

As summarized in Table 3, the included studies also varied in terms of study setting, data sources, and availability of exposure denominators, all of which influenced the interpretation of injury outcomes.

Most studies were conducted in mixed settings, capturing injuries occurring during both training and competition, although several reported a higher proportion of injuries during training than in competition. Few studies have focused specifically on competition settings, particularly at the elite or tournament level.

In terms of data sources, five studies relied on self-reported questionnaires, which may be subject to recall bias and the over-reporting of minor symptoms. In contrast, three studies employed prospective surveillance systems, including monitoring by medical staff or physiotherapists, which likely improved the diagnostic accuracy and injury classification. Two studies utilized retrospective medical record reviews, focusing on clinically diagnosed injuries but potentially underestimating minor or unreported conditions.

The availability of exposure denominators also varied between the studies. Seven studies reported injury rates relative to training hours, and two reported athlete exposure (AEs). However, some studies did not provide standardized exposure data, limiting the ability to calculate or compare the injury incidence rates.

Together, these variations in study characteristics contribute to the heterogeneity in reported injury epidemiology and should be considered when interpreting and comparing the findings across studies.

## 3 Results

### 3.1 Overview of the study characteristic and populations studied

Ten unique studies were included in this scoping review. The studies were published between 2013 and 2025 and examined injury epidemiology in badminton players across a broad developmental spectrum, with a predominant focus on children, adolescents, and elite junior players. Although some studies included wider age ranges, all provided extractable data relevant to adolescent or youth badminton players.

Most studies were conducted in Asia, particularly Japan (*n* = 6), followed by Malaysia (*n* = 1), Sri Lanka (*n* = 1), and Indonesia (*n* = 1) and one multinational (*n* = 1). No eligible studies from India were identified, showing a significant gap in region-specific injury surveillance despite the growing participation in the sport and the competitive success of Indian athletes.

The populations included elite youth national players, national-level tournament participants, pre-adolescents, adolescents, and multi-national elite junior players (aged 15–19 years) (1–3, 22). The study designs are commonly prospective or retrospective observational cohorts, with some questionnaire cross-sectional surveys used for younger players (21). All studies reported sport-specific injury outcomes, enabling the synthesis of injury types, anatomical locations, and contextual factors relevant to youth and adolescent badminton players' injuries.

### 3.2 Epidemiology of injury

The epidemiological findings presented in this review were synthesized from studies involving youth and adolescent badminton players, although some studies included broader age ranges extending into young adulthood. Where age-specific data were available, findings relevant to youth and adolescent populations were extracted and synthesized separately. However, age-stratified reporting was not consistently available across all studies, and some epidemiological estimates were derived from cohorts of individuals of mixed ages. Furthermore, substantial variability exists in injury definitions, surveillance methods, and outcome measures, which limits direct comparisons between studies. Table 5 presents the epidemiology of all included studies.

**Table 5:** Injury epidemiology.

Sr. no	Author (Year)	Key anatomical locations	Upper limb (%)	Lower mimb (%)	NECK	Trunk (%)	Abdomen	Most affected ite
1	Zhou et al. (2025) (1)	Knee, ankle, lower back, shoulder, plantar	Shoulder 7.6%	Knee-18.7% Ankle-13.4%	0–5.1%	12.3% (males); 10.0% (females)	Not reported	Ankle, Knee
2	Zhou et al. (2023) (16)	Ankle, knee, plantar, shoulder, lower back	18.20%	64.90%	2%			Ankle, knee, plantar region, shoulder, and lower back
3	Miyake et al. (2022) (19)	Racket-side (RS) shoulder/clavicle, RS thigh, lumbar spine/lower back, RS knee, non-racket side (N-RS) ankle, N-RS foot	RS shoulder/clavicle (4.35 per 1,000 AEs),	RS thigh in high school females (2.21 per 1,000 AEs)	Not reported	lumbar spine/lower back (1.83–1.25 per 1,000 AEs)	Not reported	Shoulder, thigh, lumbar spine/lower back
4	Suryanto et al. (2022) (18)	Trunk, Upper extremities, Lower extremities	29.2% (7/24 injuries)	58.3% (14/24injuries)	Not separately reported as distinct category	12.5% (3/24 injuries)	Not separately reported as distinct category	Lower extremities (58.3%)
5	Zhou et al. (2022) (21)	Shoulder, Ankle, Wrist, Knee, Groin, Elbow	Approximately 15.5% Shoulder (6.5%), Wrist (4.0%), Elbow (3.0%), Finger (2.0%)	Approximately 68.1% Ankle (19.4%), Knee (16.2%), Foot (12.4%), Hamstring (6.6%), Achilles tendon (6.1%), Groin (4.3%),	Not reported	5.3% (Lumbar)	Not specifically reported	Ankle (19.4%)
6	Senadheera et al. (2021) (22)	Head, neck, shoulder, arm, elbow, forearm, wrist, hand, trunk, back, hip, thigh, knee, leg, ankle, foot	Not reported as aggregated percentage	62.20%	Not reported as aggregated percentage	Not reported as aggregated percentage	Not reported as aggregated percentage	Lower limb, Ankle, Back injuries
7	Zhou et al. (2021) (23)	Shoulder, lower back, knee	Shoulder: 3.0%	Knee: 16.2%	Not specifically reported as separate category	Lower back: 5.3%	Not reported	Knee, shoulder, and lower back
8	Kaldau et al. (2021) (3)	Knee (*n* = 28), ankle (*n* = 19), lower back (*n* = 10)	Not separately quantified in study	knee (*n* = 28), ankle (*n* = 19)	Not reported	Lower back (*n* = 10)	Not reported	Lower extremities and lower back were predominant injured regions
9	Miyake et al. (2016) (24)	Lumbar spine, knee joint, shoulder joint	Not explicitly provided	Not explicitly provided	Not explicitly provided	Not explicitly provided	Not explicitly provided	Lumbar spine, knee joint, and shoulder
10	Goh et al. (2013) (2)	Foot, Shoulder, ankle, leg, and knee. Thigh, Gluteal, Back/Spine, Chest	1.60%	71.40%	Not reported	27.00%	Not reported	LE injury predominated

#### 3.2.1 Pain prevalence

Pain prevalence is commonly assessed using self-reported questionnaires and symptom-surveillance tools. These measures cover the pre-adolescent spectrum of musculoskeletal complaints, including minor symptoms and subclinical conditions that may not require medical attention or result in time-loss from sports participation. Consequently, studies employing symptom-based surveillance generally reported higher prevalence estimates than those using stricter injury definitions based on medical attention or time-loss (21, 22). The findings indicate that musculoskeletal pain is common among adolescent badminton players and may be an important precursor to more significant injuries.

#### 3.2.2 Injury prevalence

Injury prevalence refers to the proportion of athletes who reported injuries during a specified period. Reported prevalence estimates varied considerably across studies, reflecting differences in injury definitions, recall periods, and participant characteristics. For example, a prevalence of 33.87% has been reported among elite junior badminton players in Sri Lanka (20). Studies using broader injury definitions generally reported higher prevalence estimates than those restricting injuries to time-loss or medically attended cases. This variation highlights the influence of methodological differences on prevalence reporting and underscores the need for standardized injury surveillance in badminton.

#### 3.2.3 Injury incidence

Injury incidence has been reported using several epidemiological measures, including the incidence proportion, clinical incidence, and exposure-based incidence rates. The incidence proportion and clinical incidence describe the occurrence of new injuries in a defined population over a specific period. For instance, an incidence proportion of 18% and a clinical incidence of 18.8 injuries per 100 athletes were reported among Indonesian junior badminton players (18). Exposure-based incidence rates ranged from 0.38 injuries per 1,000 training hours in 611 players aged 7–12 years (pain-based surveillance) (21) to 5.1 injuries per 1,000 training hours in 711 national tournament-level players aged 13–18 years (time-loss and medical attention definitions) (1). Lower incidence rates have generally been reported among younger players aged 7–12 years, whereas higher rates have been observed among older adolescents and national-level athletes. Prospective and cohort studies reported an age-related increase in injury risk, with injury incidence peaking during mid-to-late adolescence (15–18 years) (1, 18, 22). This trend may be attributable to increased training volume, greater competitive demands, and physiological changes associated with growth and maturation.

#### 3.2.4 Overuse injuries

Overuse injuries are frequently reported among adolescent badminton players, particularly among older junior and elite athletes. In contrast, acute injuries are more common among younger adolescent players (24). The higher occurrence of overuse injuries among older athletes may be related to their cumulative training load, repetitive sport-specific movements, and prolonged exposure to competitive badminton. Commonly affected regions include the shoulder, knee, and lower back, reflecting the biomechanical demands of repetitive overhead strokes, jumps, lunges, and rapid changes in direction. These findings suggest that overuse injuries represent a substantial component of the injury burden in adolescent badminton players and warrant targeted preventive strategies to reduce their occurrence.

Overall, epidemiological evidence indicates that injuries in adolescent badminton players are influenced by age, exposure level, and injury surveillance methodology. Despite variability in reported estimates, the findings consistently demonstrate a high burden of musculoskeletal complaints and injuries, particularly affecting the lower extremities, with the knee and ankle being the most frequently injured sites, followed by the shoulder and lower back.

### 3.3 Lower extremity injuries

Lower extremity injuries dominate the badminton injury profile, consistently accounting for 58.3% (*n* = 128) of the reported injuries across studies (18). The knee and ankle are the most frequently affected joints in the lower limbs. The knee accounts for approximately 16%–19% of injuries or pain cases (1, 19). The most common knee and ankle injuries are attributable to the sport's demands for rapid directional changes, lunges, jumps, and landings. Across studies, lower limb injuries constituted the majority of musculoskeletal complaints, irrespective of playing level, although the proportion varied depending on the study methodology and injury definitions.

### 3.4 Upper extremity injuries

Upper extremity injuries are less frequent than lower limb injuries but remain a notable concern, particularly among elite adolescent players. Shoulder injuries are among the most frequently reported upper-extremity complaints in youth and adolescent badminton players. Across the three studies, shoulder-related problems accounted for 3% (*n* = 611), 7.6% (*n* = 711), and 18.2% (*n* = 366) of the reported injuries or pain complaints, respectively. However, direct comparisons between studies should be interpreted with caution because of differences in injury definitions, surveillance methods, and outcome measures. Studies using symptom- or pain-based surveillance tended to report a higher proportion of shoulder complaints than studies using injury-based reporting systems (1, 16, 21). The shoulder is particularly affected in female players, possibly because of differences in biomechanics and stroke technique (1).

Wrist and elbow injuries were rare and were mostly associated with repetitive smashing or overhead strokes. These injuries were predominantly overuse in nature, and few led to time-loss, suggesting that surveillance methods relying solely on medical attention definitions may underestimate their prevalence (16, 21). Overall, upper limb injuries highlight the importance of technique training, conditioning, and load management for racquet control and repetitive overhead movements. According to a study (17, 22), shoulder injuries accounted for a significant proportion of injuries among national tournament-level players, with overuse injuries such as tendinopathy being particularly common. To quantify these findings, the author reported injury rates for the racket-side (RS) shoulder/clavicle at 4.35 injuries per 1,000 AEs among female university students. Overuse injuries comprised approximately 89.7% of shoulder and clavicle injuries, indicating a strong chronic strain component in this anatomical region (17).

### 3.5 Back and trunk injuries

Back injuries, predominantly lower back pain, have been reported in multiple studies, with a prevalence ranging from 5.3% (*n* = 611) to 27% (*n* = 58) among youth and adolescent badminton players (21). A higher prevalence was observed in players who reported longer daily training durations (>2.5 h/day) or intensive training schedules. Across studies, back injuries were largely classified as overuse-related, with acute traumatic back injuries reported infrequently. Repetitive sport-specific movements, including lunges, jumps, and trunk rotation, are commonly associated with lower back symptoms. Some studies have reported a slightly higher prevalence of lower back injuries in female players than in male players, although the findings were inconsistent across the studies.

### 3.6 Overuse and acute injuries

Across the included studies, the relative distribution of acute and overuse injuries varied according to age group and competition level. Studies involving younger players (7–12 years) reported a higher proportion of acute injuries, most commonly sprains and muscle strains sustained during training or practice (2, 16, 21). In contrast, studies on older adolescents (13–18 years) and elite junior players reported a greater proportion of overuse injuries, predominantly affecting the knee, ankle, shoulder, and lower back (1, 3, 22).Studies using tournament-based surveillance have reported a higher proportion of acute injuries during competitions, whereas training exposure has accounted for a larger overall injury burden due to repetitive loading (18, 20). This distinction highlights the need for age- and training-specific injury prevention strategies, with acute injury management prioritized for younger players and overuse prevention emphasized for older players.

### 3.7 Injury risk factors

The identification of risk factors, such as age, training volume, and competition level, has been informed by studies employing varied epidemiological outcomes. For example, higher injury incidence rates in mid-to-late adolescence (18, 25) align with the observed increases in injury prevalence and symptom reports in this age group. However, differences in injury definitions and surveillance methods across studies necessitate cautious inferences regarding the risk magnitude.

#### 3.7.1 Risk factors reported in badminton studies

Several key injury risk factors were consistently reported across all 10 studies. Age and growth stage are commonly associated with injury occurrence, with a higher injury incidence observed during mid-to-late adolescence, peaking at approximately 15–16 years in males and 17–18 years in females (1, 22). Training volume was identified as a risk factor in one study (16) reported that players training more than 2.5 h per day had significantly greater odds of shoulder pain (21). A history of previous injury is associated with a greater likelihood of recurrent injuries, particularly in the lower extremities (1). Competitive level was also relevant, as elite junior players demonstrated higher injury incidence and greater injury severity than recreational or developmental players (3, 22). A study reported sex-related differences in injury patterns, with female players demonstrating a higher prevalence of shoulder and lower back injuries, whereas male players more frequently sustained ankle and knee injuries. However, the remaining included studies did not consistently report sex-stratified injury outcomes, limiting the ability to draw broader conclusions regarding sex-specific injury patterns in youth and adolescent badminton players (1). In addition, several extrinsic factors, including the training environment and technique, such as hard court surfaces, inappropriate footwear, and suboptimal stroke mechanics, have been proposed as potential contributors to injury risk in youth and adolescent badminton players (2, 26).

#### 3.7.2 Broader youth sports evidence relevant to badminton (contextual evidence)

To contextualize these findings, evidence from the broader adolescent sports injury literature supports the involvement of several factors in the nature of the injury risk. Age and growth stage are critical, with injury incidence peaking during mid-to-late adolescence (approximately 15–16 years for males and 17–18 years for females). Rapid skeletal growth increases susceptibility to overuse and stress injuries (27). Training volume has a significant impact on injury risk. Training for more than 2 h a day, having high weekly training hours, and focusing on one sport increase both overuse and sudden injuries (25, 27). Excessive training without adequate rest can lead to biomechanical breakdowns. Previous injuries increase the risk of recurrent injuries, particularly in the lower limbs, by impairing biomechanical function (24). Elite junior players have higher injury rates than recreational players because of increased competition (28). Sex differences exist, as females show higher shoulder and back injuries, whereas males have more ankle and knee injuries than females. Female players have nearly a 10% risk of anterior cruciate ligament injury during high school. Male and female adolescents in soccer, basketball, lacrosse, and football showed particular injury risk (29, 30). Insufficient warm-up, lack of preparation, and poor supervision can lead to a higher chance of injury. Adolescent players who get less than eight hours of sleep are 1.7 times more prone to injuries (31). Injury risk involves both intrinsic (age, sex, and injury history) and extrinsic (training volume, competition level, and environment) factors. Prevention requires addressing these issues through targeted programs, monitoring, rest, and proper instruction (24, 27, 29, 32). Together, these risk factors can be categorized as intrinsic (age, sex, growth stage, and prior injury) and extrinsic (training volume, specialization, competition level, equipment, and environment), several of which are modifiable through structured prevention strategies.

## 4 Discussion

This scoping review synthesized evidence from 10 studies examining injury epidemiology, injury patterns, and risk factors among youth and adolescent badminton players. The findings indicate a substantial burden of musculoskeletal injuries, although direct comparisons across studies are challenging because of differences in injury definitions, surveillance methods, and exposure measurements (18, 22, 24, 33).

A consistent finding across studies is the increase in injury occurrence during mid-to-late adolescence, which may be attributed to increased training volumes, greater competitive demands, and growth-related physiological changes (18). Younger athletes were more likely to sustain acute injuries, whereas overuse injuries were more commonly reported among older adolescents and elite junior players (23, 24).

Lower extremity injuries represented the predominant injury burden across the included studies, with the ankle and knee being the most frequently affected anatomical regions (18, 25, 27). These findings are consistent with the biomechanical demands of badminton, which involve repetitive lunging, jumping, rapid deceleration, and multidirectional movement patterns (6, 19). The lumbar spine and shoulder were also frequently affected, particularly among older adolescents and athletes exposed to greater training loads (22, 25, 33). Upper extremity injuries, including wrist and elbow conditions, are reported less frequently but remain clinically relevant because of the repetitive overhead and racket-specific movements required in badminton (23, 33).

Age- and sex-related differences in injury patterns were also observed. Injury incidence generally increases during mid-to-late adolescence, coinciding with periods of rapid growth, increased training volume, and greater competition demands (18). Sex-specific peaks in injury incidence were identified, with males demonstrating peak injury rates between 15 and 16 years of age and females between 17 and 18 years of age. These findings suggest a potential interaction between biological maturation, training exposure, and sports-specific requirements. Furthermore, several studies reported developmental shifts in injury patterns, with younger players more frequently sustaining lower extremity injuries, whereas older adolescents exhibited greater involvement of the lumbar spine and shoulder (1, 22, 25, 33). Collectively, these findings highlight the importance of considering growth- and maturation-related factors when developing injury prevention and training programs for young badminton athletes.

Training exposure and early sport specialization emerged as important modifiable risk factors. Studies have reported that injuries occur more frequently during training than during competition, emphasizing the cumulative effects of repetitive loading (24, 25, 27). Pain- and symptom-based studies have further suggested that musculoskeletal complaints may develop before clinically significant or time-loss injuries occur, highlighting the importance of early symptom monitoring and intervention (1).

Despite the growing popularity of badminton worldwide, several gaps in the literature still remain. Most studies have focused on elite or national-level athletes, with limited evidence available for community-level and sub elite athletes. In addition, the lack of standardized injury definitions, severity classifications, and exposure reporting methods limits the comparability of the studies (20). Longitudinal investigations examining the cumulative effects of growth, maturation, training load, and specialization are scarce. Furthermore, despite increasing participation and international success, epidemiological research in regions such as India remains limited.

Collectively, these findings highlight the need for comprehensive injury prevention strategies targeting the modifiable risk factors. Age-appropriate load management, neuromuscular training, movement quality interventions, and structured recovery programs may help reduce the risk of injury during critical developmental periods (10, 20). At an organizational level, the implementation of standardized injury surveillance systems within youth badminton programs would facilitate injury monitoring, improve research comparability, and support the development of evidence-based prevention initiatives.

## 5 Clinical implications and prevention strategies

The findings of this review indicate that musculoskeletal injuries and pain complaints are common among youth and adolescent badminton players, particularly affecting the lower extremities, lumbar spine, and shoulder regions. Several studies identified age, training exposure, and competitive level as potential factors associated with injury occurrence. Injury rates appeared to increase during adolescence, highlighting the importance of age-appropriate training, workload monitoring, and adequate recovery strategies.

Regular monitoring of musculoskeletal symptoms may facilitate early identification of injury risk and support timely intervention. Education of athletes, coaches, parents, and support personnel regarding workload progression, symptom recognition, and recovery practices may contribute to reducing injury burden. Furthermore, the adoption of standardized injury surveillance systems in youth badminton programs may improve injury monitoring and support the development of evidence-informed prevention strategies.

## 6 Strengths and limitations

This review provides a comprehensive overview of injury epidemiology, patterns, and risk factors among youth and adolescent badminton players. The strengths of this study include the use of a structured scoping review methodology, adherence to the PRISMA-ScR guidelines, and the inclusion of studies from diverse countries and competitive levels.

This study has several limitations. Substantial heterogeneity in injury definitions and surveillance methods across studies limits the comparability of the findings and reduces the strength of the conclusions regarding injury incidence and risk factors. For example, the wide range in reported injury incidence rates (0.38–5.1 per 1,000 training hours) reflects methodological differences rather than true variation in injury risk, and direct comparisons should be avoided. Some studies included participants outside the target age range, and age-specific findings were not always reported. Only English-language studies were included, which may have introduced a language bias. Despite high badminton participation in China and Indonesia, no eligible studies from these countries were identified in the English-language literature. This limits the generalizability of the findings to these regions, where training practices, injury patterns, and risk factors may differ from those reported in the studies included in this review. Additionally, study screening and data extraction were conducted by a single reviewer without independent verification, which deviates from the recommended best practices for scoping reviews and may have increased the risk of selection or extraction errors. To mitigate this, predefined eligibility criteria were applied consistently, and ambiguous cases were discussed with co-authors before final inclusion, and no formal methodological quality appraisal was performed, which is consistent with the objectives of a scoping review.

## 7 Conclusion

This scoping review highlights the substantial burden of musculoskeletal injuries among youth and adolescent badminton players, with overuse injuries affecting the lower extremities, lumbar spine, and shoulders most frequently. Injury occurrence generally increases during adolescence and with greater training exposure, reflecting the combined influence of growth, maturation, and repetitive, sport-specific loading. Despite the growing interest in badminton injury research, substantial heterogeneity in injury definitions, surveillance methods, and exposure measurements limits comparisons across studies. Future research should prioritize standardized injury surveillance, consistent exposure-based reporting, and longitudinal study designs to improve the understanding of injury mechanisms and support the development of evidence-based injury prevention strategies for youth and adolescent badminton players.
